# Psychiatric disorders and violent reoffending: a national cohort study of convicted prisoners in Sweden

**DOI:** 10.1016/S2215-0366(15)00234-5

**Published:** 2015-09-02

**Authors:** Zheng Chang, Henrik Larsson, Paul Lichtenstein, Seena Fazel

**Affiliations:** Department of Psychiatry, Warneford Hospital, University of Oxford, Oxford, UK (Z Chang PhD, Prof S Fazel MD); and Department of Medical Epidemiology and Biostatistics, Karolinska Institutet, Stockholm, Sweden (Z Chang, H Larsson PhD, Prof P Lichtenstein PhD)

## Abstract

**Background:**

Reoffending and presence of psychiatric disorders are common in prisoners worldwide. However, whether psychiatric disorders are risk factors for reoffending is still unknown. We aimed to examine the association between psychiatric disorders, including substance use disorder, and violent reoffending.

**Methods:**

We did a longitudinal cohort study of 47 326 prisoners who were imprisoned since Jan 1, 2000, and released before Dec 31, 2009, in Sweden. We obtained data for diagnosed psychiatric disorders from both inpatient and outpatient registers, and sociodemographic and criminological factors from other population-based registers. We calculated hazard ratios (HRs) for violent reoffending with Cox regression. To control for potential familial confounding, we compared sibling prisoners with and without psychiatric disorders. We calculated population attributable fraction to assess the population effect.

**Findings:**

Diagnosed psychiatric disorders were associated with an increased hazard of violent reoffending in male (adjusted HR 1·63 [95% CI 1·57–1·70]) and female (2·02 [1·54–2·63]) prisoners, and these associations were independent of measured sociodemographic and criminological factors, and, in men, remained substantial after adjustment for unmeasured familial factors (2·01 [1·66–2·43]). However, findings differed between individual diagnoses and sex. We found some evidence of stronger effects on violent reoffending of alcohol and drug use disorders and bipolar disorder than of other psychiatric disorders. Alcohol use disorder seemed to have a greater effect in women than in men (women 2·08 [1·66–2·60]; men 1·63 [1·56–1·71]). The overall effects of psychiatric disorders did not differ with severity of crime. The hazard of violent reoffending increased in a stepwise way with the number of diagnosed psychiatric disorders. Assuming causality, up to 20% (95% CI 19–22) of violent reoffending in men and 40% (27–52) in women was attributable to the diagnosed psychiatric disorders that we investigated.

**Interpretation:**

Certain psychiatric disorders are associated with a substantially increased hazard of violent reoffending. Because these disorders are prevalent and mostly treatable, improvements to prison mental health services could counteract the cycle of reoffending and improve both public health and safety. National violence prevention strategies should consider the role of prison health.

**Funding:**

Wellcome Trust, Swedish Research Council, and Swedish Research Council for Health, Working Life and Welfare.

## Introduction

More than 10 million people are currently in prison worldwide,^1^ and substantially larger numbers of ex-prisoners are living in society.^2^ Despite reported decreases in violence in many countries,^3^ repeat offending remains high across many high-income and middle-income countries.^4^ In the USA and UK, more than a third of released prisoners are reconvicted for a new crime within 2 years, and more than half within 5 years.^5,6^ Furthermore, about 70% of those convicted in the USA are repeat offenders.^7^ In England and Wales, this figure is estimated at 90%,^8^ and the proportion of individuals convicted who have had 15 or more previous offences has been increasing since 2008.^9^

Much research has focused on identification of individuals at high risk of reoffending. Although a substantial amount is known about demographic risk factors for reoffending,^10–12^ uncertainty remains about its mental health determinants.^13^ Research specifically related to reoffending is different from that in the general population because in the general population, several psychiatric disorders have been shown to be associated with an increased risk of committing and conviction for violence and violent crime,^14–16^ whereas in offenders, this association is not consistent. Because psychiatric disorders are prevalent and mostly treatable, with some studies suggesting that one in seven prisoners has a psychotic illness or major depression, and about one in five people enter prison with clinically significant substance use disorders,^17^ tackling them has the potential to substantially reduce adverse outcomes in released prisoners. In the USA, for example, estimates suggest that 15% of prisoners have a severe mental illness,^18^ and the number of individuals with mental illness in prisons and jails is ten times that in public psychiatric hospitals.^19^

The little research into psychiatric disorders and reoffending that has been done has led to divergent findings. Authors of systematic reviews with heterogeneous samples^10,20^ have concluded that psychosis is inversely related to reoffending. By contrast, authors of a focused review^13^ reported that psychosis increased risk of reoffending, although it was only based on four studies that used control groups without psychiatric disorder. However, even in these investigations, causality has not been shown, and several potential confounders have not been fully examined.^13^ First, whether this association is attributable to sociodemographic and criminological factors is uncertain.^21^ Second, findings from some studies suggest that the association is mainly due to substance misuse,^22–24^ and whether other common psych iatric disorders are independently related to risk of reoffending needs further examination. Third, although both criminal activity^25^ and most psychiatric disorders^26^ have long been known to run in families, the contribution of familial (genetic and early environmental) factors to the association has not been investigated. Finally, few studies have been done on female prisoners, who have higher prevalences of psychiatric disorders than do men in prison.^17^

In this population-based longitudinal study of released prisoners, we aimed to investigate the association between psychiatric disorders and violent reoffending and to address three questions. First, whether being diagnosed with any psychiatric disorder is independently associated with violent reoffending. Second, whether this association differs by psychiatric diagnosis. Finally, whether this association is explained or moderated by comorbid substance use disorder. We did the analyses by controlling for sociodemographic and criminological factors, but also comparing sibling prisoners with and without psychiatric disorder, a powerful approach to control for familial confounding.

## Methods

### Study setting

We linked the following population-based registers in Sweden: the National Crime Register, which includes detailed information about all criminal convictions since 1973; the National Patient Register, which provides diagnoses for all inpatient psychiatric hospital admissions since 1973 and outpatient care since 2001; the Migration Register, which supplies information about dates of migration into or out of Sweden; the Cause of Death Register, which contains information about dates and causes of all deaths since 1958; the Multi-Generation Register, which contains information about biological relationships for all individuals living in Sweden since 1933; and the Longitudinal Integration Database for Health Insurance and Labour Market studies, which contains yearly assessments of income, marital and employment status, and education for all individuals aged 16 years or older since 1990.

In Sweden, all residents (including immigrants) have a unique personal identifier used in all national registers, thus enabling data linkage.^27^ We selected a cohort of all convicted prisoners who have been imprisoned since Jan 1, 2000, and released before Dec 31, 2009. All individuals were followed up from the day of release until first reoffence of violent crime, death, emigration, or end of the study (Dec 31, 2009). We identified prisoners with full siblings using the Multi-Generation Register. This study was approved by the Regional Ethics Committee at the Karolinska Institutet (Stockholm, Sweden).

### Measures

We linked prisoners within the study cohort to the National Patient Register to obtain information about diagnosed psychiatric disorders. We identified those with any lifetime psychiatric diagnoses (based on the ICD Eighth [ICD-8; code 290–315], Ninth [ICD-9; code 290–319], and Tenth [ICD-10; code F00–F99] Revisions) before release from prison. To explore the difference between individual disorders and the effect of comorbidity, we investigated the following specific psychiatric disorders: alcohol use disorder (ICD-8: 291 and 303; ICD-9: 291, 303, and 305A; ICD-10: F10), drug use disorder (ICD-8: 304; ICD-9: 292, 304, and 305 [except .A]; ICD-10: F11–F19), personality disorder (ICD-8: 301 [except .1]; ICD-9: 301 [except .B]; ICD-10: F60–F61), attention-deficit hyperactivity disorder (ICD-8: not applicable; ICD-9: 314; ICD-10: F90), and other developmental or childhood disorders (ICD-8: 308; ICD-9: 299A, 312, 313, and 315; ICD-10: F80–F98 [except F90]).

We assigned a hierarchical approach to differentiate between schizophrenia spectrum disorders, bipolar disorder, depression, and anxiety disorder.^28^ We included any individual with one of the schizophrenia spectrum disorder diagnoses, including schizoaffective and delusional disorders (ICD-8: 295, 297, 298·1–9, and 299; ICD-9: 295, 297, 298 [except .A], and 299; ICD-10: F20–F29); one of the bipolar diagnoses (ICD-8: 296.1, 296.3, 296.8, 296A, 296C–296E, and 296W; ICD-10: F30–F31), but not schizophrenia spectrum disorders; one of the depression diagnoses (ICD-8: 296.2, 296.9, 298.0, and 300.4; ICD-9: 296B, 296X, 298A, 300E, and 311; ICD-10: F32–F39), but without schizophrenia spectrum or bipolar disorder; and one of the anxiety diagnoses (ICD-8: 300 [except .4], 305, and 307; ICD-9: 300 [except .E], 306, 308, and 309; ICD-10: F40–F48), but without schizophrenia spectrum or bipolar disorder or depression. Use of Swedish national registers for psychiatric research is well established, and the patient registry data have good to excellent validity for a range of psychiatric disorders.^29–33^ Overall, the positive predictive value has been reported to be 85–95% for most diagnoses.^34^

The main outcome was any conviction of violent crime after release. In keeping with previous work, we defined violent crime as homicide, assault, robbery, arson, any sexual offence (rape, sexual coercion, child molestation, indecent exposure, or sexual harassment), illegal threats, or intimidation.^33,35^ If no date of the crime was recorded, we used the date of conviction.

Measured covariates were sex, age, immigration status (defined as being born outside Sweden), criminological factors (length of incarceration [categorised into four levels], violent index offence, and any previous violent crime), and sociodemographic factors (civil status [categorised into four levels], employment, highest level of completed education [categorised into three levels], disposable income, and neighbourhood deprivation) at the year of release. For all analyses, we investigated the index offence, which is the most serious offence that led to the prison sentence. We did not replace missing data by imputation or other methods because this imputation needs some assumptions to be made and the number of individuals with missing values was quite small, but in a sensitivity analysis, we recalculated the results with missing values imputed.

### Statistical analysis

To explore the association between psychiatric disorders and risk of violent reoffending, we compared prisoners with and without a psychiatric disorder. We used Kaplan-Meier survival curves to show the timing of violent reoffending after release from prison. To quantify the association, we used the Cox proportional hazards model, and estimated hazard ratios (HRs) in three models. In the first model, we adjusted for age and immigration status. In the second, we also adjusted for socio demographic and criminological factors. In the third, we also used sibling comparison to adjust for possible familial confounding.^36^ We did this familial adjustment by fitting a fixed-effect model^37^ (stratified Cox regression) to the subsample of same-sex full sibling prisoners. This model adjusts for all unmeasured genetic and environ mental factors that are shared by siblings, and also included the measured covariates adjusted for in models 1 and 2. We stratified all analyses by sex. We verified the proportional hazards assumption by visually checking the Kaplan-Meier curves and tested it using Schoenfeld residuals.^38^

To explore the association between each individual psychiatric disorder and risk of violent reoffending, we constructed Cox regression models for each of the diagnoses investigated. We calculated HRs in three models, with progressive adjustment for age and immigration status, sociodemographic and criminological covariates, and alcohol and drug use disorders. We further examined whether the association between psychiatric disorder and violent reoffending was moderated by substance use disorder (defined as diagnoses of alcohol or drug use disorders). We used a likelihood ratio test to examine the interaction between psychiatric disorder and substance use disorder (with p<0·05 indicating a significant interaction). Additionally, we analysed the moderating effect of substance use disorder on schizophrenia spectrum disorders and bipolar disorder. These diagnoses had the best diagnostic validity in our sample. Because comorbidity is common, we further examined the association between the number of diagnosed psychiatric disorders and violent reoffending.

To assess the population effect of psychiatric disorder on violent reoffending, we used the population attributable fraction (PAF). The PAF measures the proportion of violent reoffending in the population that can be attributed to psychiatric disorder, assuming that a causal relation exists. In the presence of confounding, PAF can be calculated as Pr(X=1∣Y=1)(1 – HR_α_^−1^),^39^ where Pr(X=1∣Y=1) is the probability of exposure given outcome and HR_α_^−1^ is the adjusted HR. We calculated confidence intervals for PAFs using the Bonferroni inequality method.^40^

To test whether the association between psychiatric disorders and violent reoffending was different depending on type of crime, we did sensitivity analyses using different outcomes. First, we restricted the outcome to specific crimes for which interpersonal violence is known to have occurred, including homicide and attempted homicide, all forms of assault (including aggravated, and assault of an officer), rape, sexual coercion, and child molestation. Second, we examined the association with other violent crime: arson, indecent exposure, sexual harassment, illegal threats, and intimidation. This breakdown also provides a proxy for testing of associations by severity of violent crime. We also examined the association between individual psychiatric disorders and these two subgroups of violent reoffending in male prisoners.

### Role of the funding source

The funders of the study had no role in study design, data collection, data analysis, data interpretation, or writing of the report. ZC had full access to all the data in the study and, with SF, had final responsibility for the decision to submit for publication.

## Results

We identified 47 326 prisoners during the study period (43 840 male and 3486 female prisoners), who we followed up for 10 years after release from prison. Baseline sociodemographic and criminological information and psychiatric diagnoses, and follow-up data in male and female prisoners are presented in table 1, and their associations with violent reoffending are presented in the appendix (pp 3–6). In male prisoners, 18 563 (42%) of 43 840 had been diagnosed with at least one psychiatric disorder before release, and 10 884 (25%) reoffended for violent crimes during follow-up. In female prisoners, a higher proportion (2233 [64%] of 3486) had been diagnosed with psychiatric disorder than had male prisoners, and fewer (379 [11%]) reoffended for violent crimes than did male prisoners. 11 804 (57%) of prisoners with psychiatric disorders had both inpatient and outpatient diagnoses (10 669 [57%] men and 1135 [51%] women). Types of violent reoffending are presented in the appendix (p 2); the most common category was assault (7171 [64%] of 11 263 individuals reoffending for a violent crime), followed by threats and intimidation, robbery, sexual offences, and homicide.

**Figure 1:**
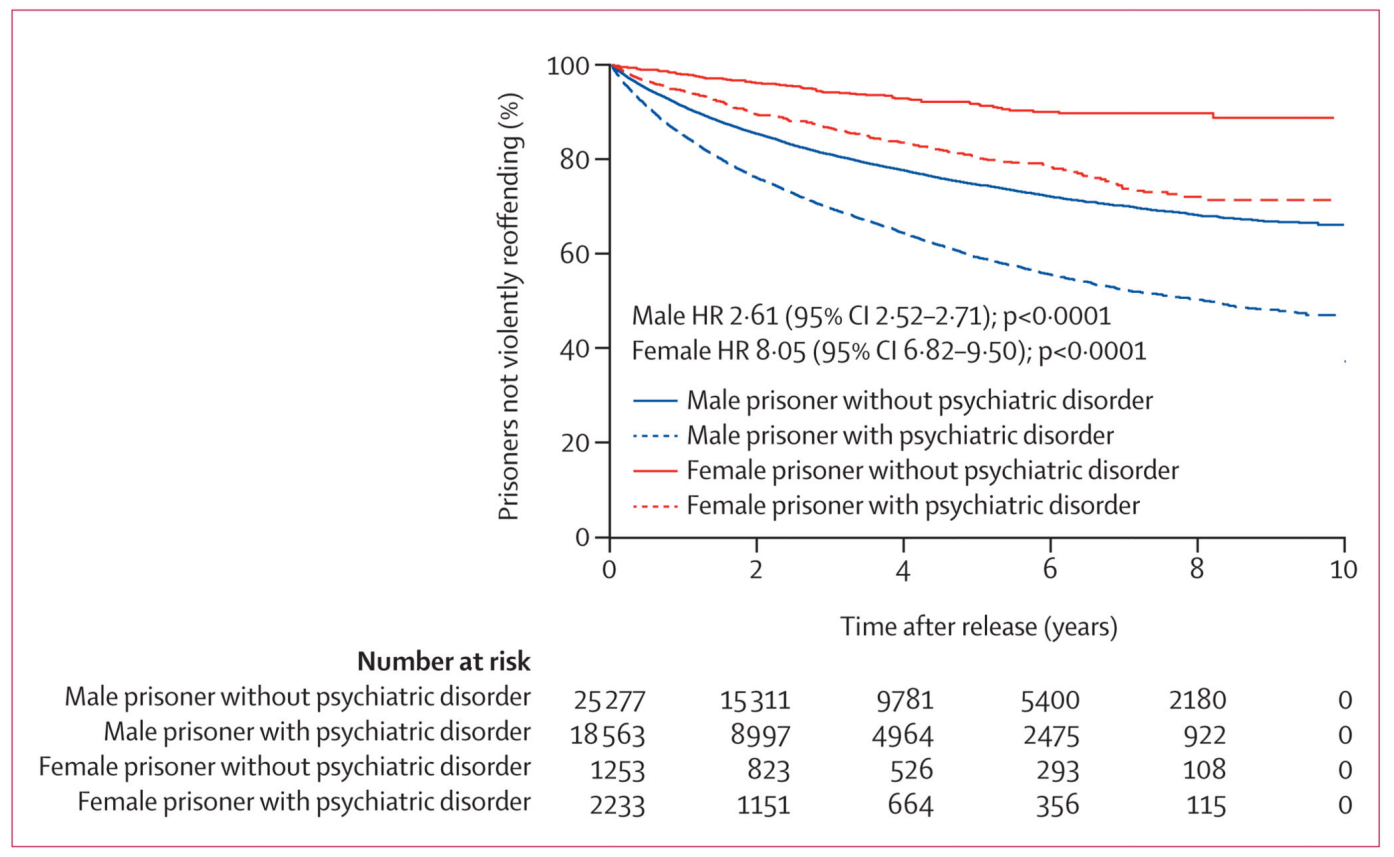
Kaplan-Meier curve (unadjusted model) for violent reoffending in released prisoners by sex and psychiatric disorder status HR=hazard ratio.

**Figure 2:**
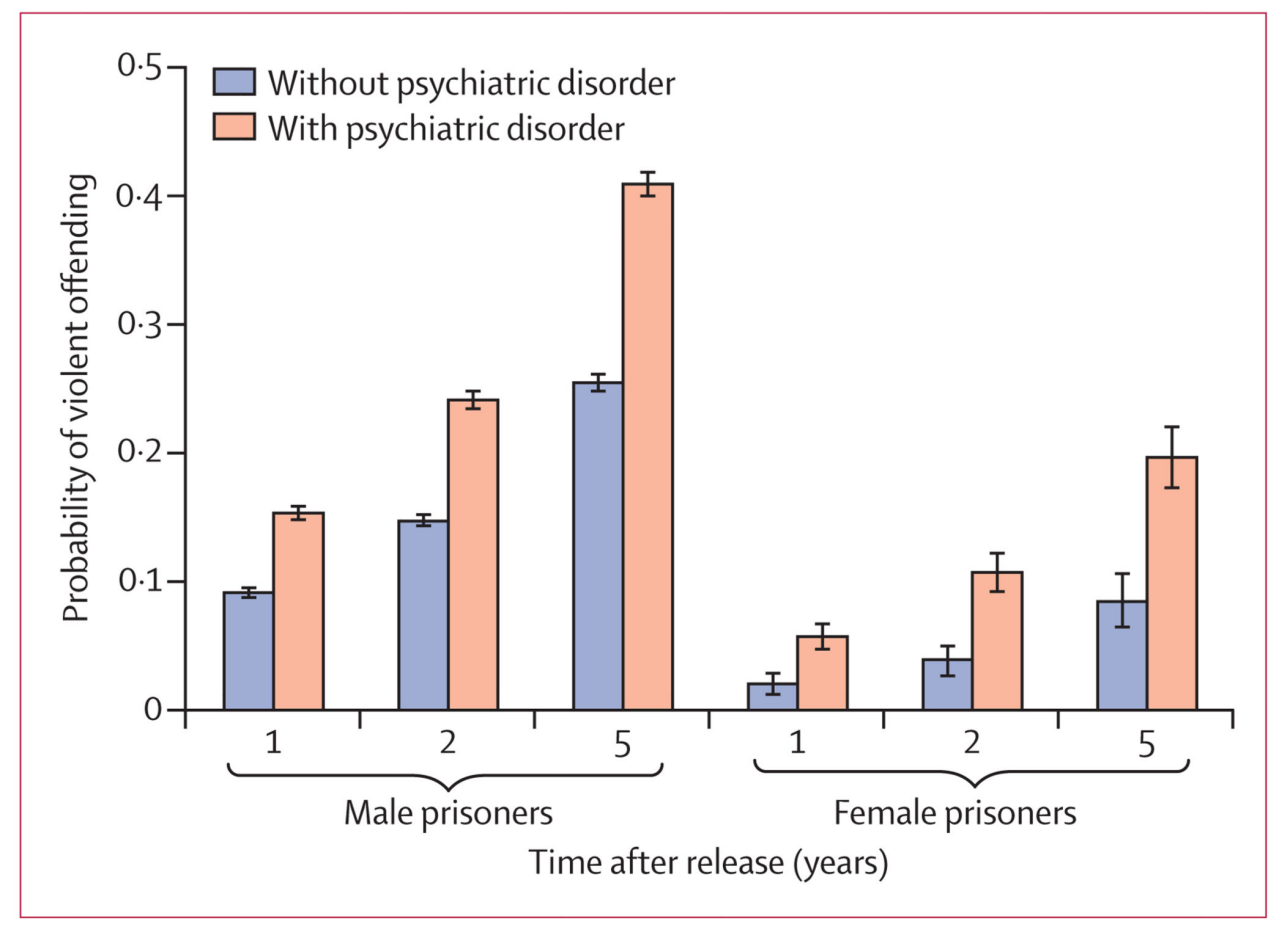
Probability of violent reoffending by sex, time after release, and psychiatric disorder status Error bars are 95% CIs.

The overall Kaplan-Meier curve for violent reoffending in released prisoners is presented in the appendix (p 1). Prisoners with any psychiatric disorder had a higher rate of violent reoffending than did those without a disorder (figure 1). In male prisoners, the median time to first violent reoffending was 2·4 months shorter for those with psychiatric disorder (median 14·2 [IQR 5·1–31·8]) than with those without (16·6 [6·2–35·2]). In female prisoners, time to violent reoffending was 4·8 months shorter for those with psychiatric disorder (18·4 [6·0–38·3]) than with those without (23·2 [10·3–41·5]). Prisoners with psychiatric disorder had a high probability of violent reoffending (figure 2): over 5 years, the probability was 0·41 (95% CI 0·40–0·42) for male prisoners with psychiatric disorder and 0·25 (0·25–0·26) for those without. In female prisoners, violent reoffending probabilities were 0·20 (0·17–0·22) for those with psychiatric disorder and 0·08 (0·06–0·10) for those without.

Cox regression analysis showed that, in male prisoners, psychiatric disorder was associated with an increased hazard of violent reoffending (model 1: HR 2·10 [95% CI 2·02–2·19]; table 2). The association was attenuated but remained substantial after adjustment for sociodemographic and criminological factors (model 2: 1·63 [1·57–1·70]). We further compared prisoners who were full siblings, and psychiatric disorder was still associated with an increased hazard of violent reoffending (model 3: 2·01 [1·66–2·43]). In female prisoners, psychiatric disorder was also associated with a higher hazard of violent reoffending (model 1: 2·76 [2·15–3·55]), and after adjustment (model 2: 2·02 [1·54–2·63]). However, the association was non-significant in the sibling model, with wide confidence intervals. We recorded similar results when analysing all siblings of prisoners (including non-prisoner siblings, appendix p 7). We also found similar results in young and adult men (appendix p 8). Even in the most adjusted model, our data provide sufficient events per variable (EPV; men: 602 EPV [10 844 events per 18 variables]; women 21 EPV [379 events per 18 variables]; 20 is deemed a sufficient number of EPV).^41,42^

10 884 incidents of violent reoffending occurred in male prisoners after release. Of these, 2187 were potentially attributable to psychiatric disorder. This corresponds to a PAF of 20% (95% CI 19–22). In female prisoners, 152 of 379 incidents of violent reoffending were potentially attributable to psychiatric disorder, with a corresponding PAF of 40% (27–52).

When we explored individual psychiatric disorders, all diagnoses were associated with an increased hazard of violent reoffending, even after adjustment for possible confounders (except for schizophrenia spectrum disorders in women in model 3), but the magnitude of associations varied and some hazards were not significantly increased in women (table 3). We found the strongest associations for alcohol and drug use disorders, personality disorder, attention-deficit hyperactivity disorder, other developmental or childhood disorders, schizophrenia spectrum disorders, and bipolar disorder.

Because of the small sample size of female prisoners, we did the following analyses in male prisoners only. The proportion of male prisoners who violently reoffended and had any psychiatric disorder along with substance use disorder comorbidity was higher than in those without this comorbidity, and the adjusted HR was also higher (table 4). A test of interaction between any psychiatric disorder and substance use disorder was not significant. We noted similar results for schizophrenia spectrum and bipolar disorder.

**Figure 3:**
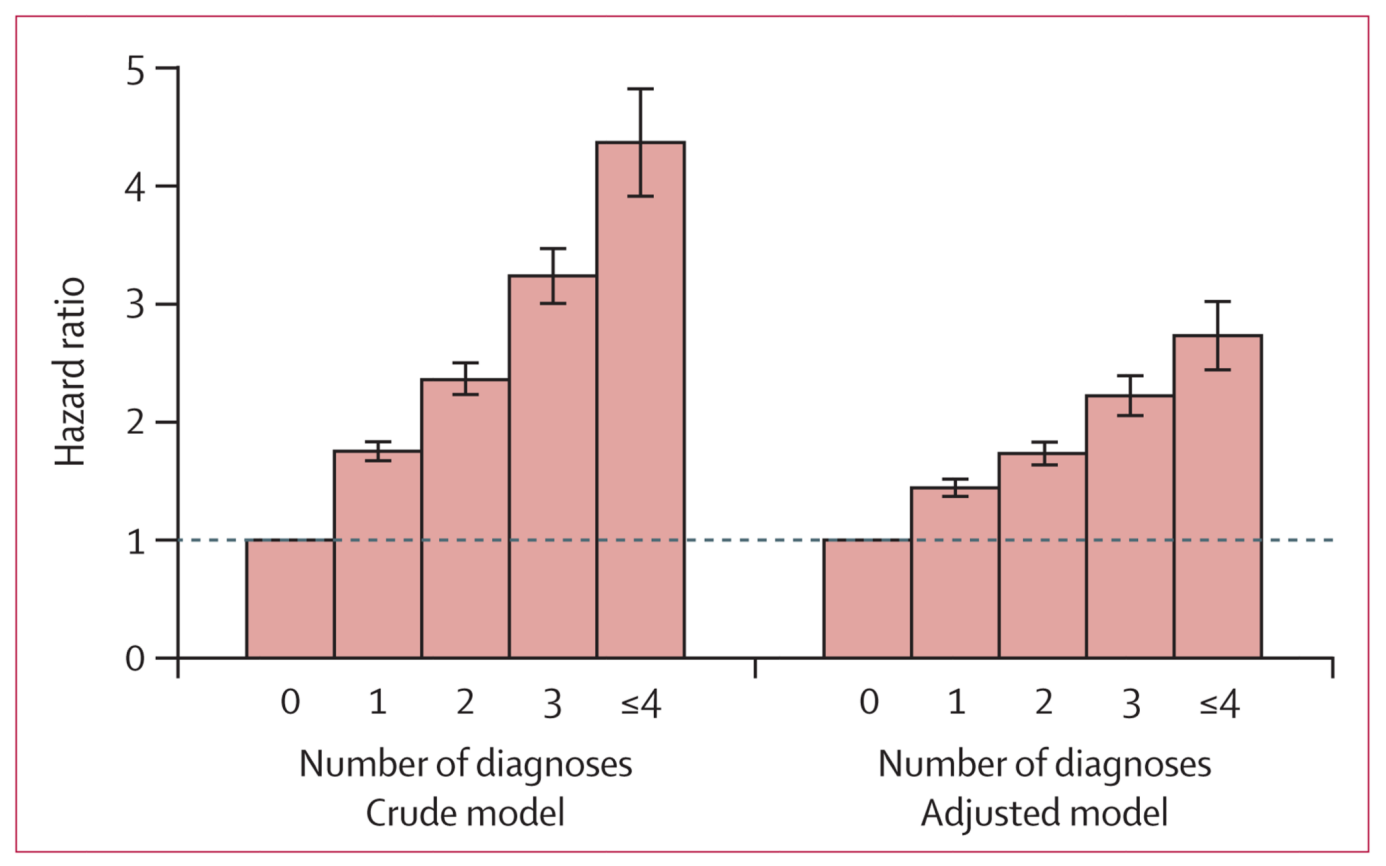
Association between number of psychiatric disorders and violent reoffending in male released prisoners Error bars are 95% CIs.

The hazard of violent reoffending increased in a stepwise way according to the number of psychiatric disorders (figure 3). Individuals with four or more psychiatric disorders had a substantially increased hazard of reoffending compared with those without psychiatric disorder (adjusted HR 2·74 [95% CI 2·45–3·06]). In sensitivity analyses (appendix pp 9–11), our findings did not differ when we restricted outcomes to interpersonal violent crimes or other violent crimes, or used imputed samples.

## Discussion

In this longitudinal study, we have shown that psychiatric disorders were associated with a substantially increased hazard of violent reoffending. The association was independent of a number of measured sociodemographic, criminological, and familial factors, except that the finding in female prisoners was non-significant when taking familial factors into account. To our knowledge, we are the first to use a sibling design to study reoffending in an unselected prison population. Additionally, with important caveats, we have estimated the population impact of psychiatric disorders on violent reoffending.

Our study has three main findings. First, any diagnosed psychiatric disorder was associated with a substantially increased hazard of violent reoffending; however, the hazard ratio decreased after adjustment for sociodemographic and criminological factors, suggesting that about 40% of the excess violent reoffending was due to these factors. More importantly, this result suggests that psychiatric disorders (which included both inpatient and outpatient diagnoses) were associated with an increased hazard of violent reoffending independently of these factors. This finding is by contrast with the findings of systematic reviews,^10,20^ some expert opinion,^21^ and scores assigned to mental disorders in widely used risk assessment instruments in criminal justice.^43^ But it is in keeping with a few cohort investigations, although these studies have used small numbers of prisoners^44^ or selected samples of high-risk prisoners^45^ or community offenders.^46^ Furthermore, our findings are consistent with those from a large retrospective study in the USA,^47^ which showed that psychiatric disorders are associated with increased hazard of previous incarcerations. However, because of the retrospective design, investigators of this study were unable to show a temporal sequence between exposure and outcome, and exclude the possibility of reverse causation.

Additionally, we noted no evidence of familial confounding on the association between psychiatric disorder and violent reoffending in men, but in women, adjustment for familial confounding made the finding non-significant. The temporality between measures of psychiatric disorders and violent reoffending, and the gradient effect of number of diagnoses on reoffending provide additional corroboration for a causal hypothesis.^48^ However, for causality to be clearly shown, these findings will need validation in other released prisoner cohorts and treatment trials will need to be done.

To our knowledge, we calculated PAFs to estimate the population impact of diagnosed psychiatric disorders on violent reoffending for the first time. PAFs assume causality, so our estimates should be interpreted with much caution. Additionally, because we did not have reliable information about all possible covariates and thus could not include them in our models, the reported PAFs are likely to be overestimates. Diagnostic comorbidities (such as personality disorder) and social factors that co-occur with psychiatric disorders (including victimisation and homelessness) will probably reduce our PAF estimates. Generalisation to other countries with different criminal cultures should not be made without further research. Nevertheless, the PAF that we report shows a substantial contribution of psychiatric disorder to the high risk of reoffending. In some countries, this contribution to reoffending will also be important from a public health perspective in terms of absolute numbers of crimes. For example, in the USA, former prisoners account for an estimated 15–20% of all adult arrests,^7^ so even a small PAF would lead to substantial decreases in violent crimes from the 1·1 million committed in the USA in 2013.^49^ National violence prevention strategies, which have not included prison health in their targets, strategies, or surveillance,^3^ need review on the basis of our findings.

In line with previous research,^17,50^ we noted that a higher proportion of female prisoners had psychiatric disorders than did male prisoners. The hazard ratio for violent reoffending seemed to be higher in women prisoners than in men released from prison, although the absolute rate of violent reoffences were lower in women than in men. These findings are consistent with other research that shows that women with schizophrenia and related disorders have a higher relative risk of violence than do men with these disorders,^15^ and might be attributable to women who offend being more severely psychiatrically ill than are men who offend.^51^

The second main finding was that each individual psychiatric disorder was associated with a modest increased hazard of violent reoffending. This result was unexpected, particularly in men, for whom we found similar HRs for alcohol and drug use disorders, personality disorder, attention-deficit hyperactivity dis order, other developmental or childhood disorders, schizophrenia spectrum disorders, and bipolar disorder. This finding contrasts with studies in the general population showing substance use disorder to be associated with a higher risk of violent crime than are other psychiatric disorders (particularly if they are not comorbid with substance use disorder).^52,53^ A theoretical explanation for the nonspecificity that we report could be that psychiatric disorders share core psychopathological features,^54^ such as emotional dysregulation, which increase the risk of violence.

The magnitude of the associations varied. Bipolar disorder was associated with a higher risk of violent reoffending in the familial adjusted model (model 3) than were other psychiatric disorders, apart from alcohol or drug use disorders, similar to a register-based US study.^47^ Prison health services have not focused on screening or treatment of bipolar disorder specifically, and replication of this finding and possible associations with severity and psychotic symptoms of the illness need further investigation. In women, we noted some evidence of heterogeneity by individual disorder, and the effect of alcohol use disorder seemed to be stronger than that of other psychiatric disorders. Additionally, the effect of alcohol use disorder seemed stronger in women than in men. Possible differences between various offender categories and types of violent reoffending should be considered (appendix pp 10–11). Although the overall effect of any psychiatric disorder was not materially different when different types of violent reoffending were investigated, whether affective disorders (eg, depression, bipolar disorder, and anxiety disorder) are associated with higher hazards of reoffending for less severe violent crimes than for more severe violent crimes needs further research.

The third main finding was that the association between psychiatric disorders and violent reoffending was not fully attributable to substance use disorder. In line with previous studies,^22,23,33^ we found that prisoners with severe mental illness (eg, schizophrenia spectrum disorders and bipolar disorder) and comorbid substance use disorder had a higher risk of violent reoffending than did those without comorbidity. However, we also showed that severe mental illness increased the hazard of violent reoffending, even without substance use disorder comorbidity (although this finding was not significant for bipolar disorder because of small numbers of patients with the disorder). Additionally, we found that the hazard of violent reoffending increased in a stepwise way according to the number of psychiatric disorders, and prisoners with multimorbidity of psychiatric disorders had a substantially increased risk of violent reoffending. These findings suggest that management of prisoners with psychiatric disorders should not merely focus on treatment of one disorder, but consider comorbidity and multimorbidity. The roles of antipsychotics,^55^ mood stabilisers,^55^ attention-deficit hyperactivity disorder medications,^56^ and psychological treatments in reduction of risks of repeat offending need investigation.

A limitation of our study is reliance on data from patient registers for ascertainment of psychiatric diagnoses. Although these data have good diagnostic validity and the advantage of not relying on patient recall and self-report, the prevalence of some psychiatric disorders was underestimated. The prevalence of severe mental illness was similar to the pooled prevalence in a systematic review^17^ of more than 100 studies (eg, pooled prevalence of psychosis of 3·6% in male prisoners and 3·9% in female prisoners in the systematic review *vs* 3% in male prisoners and 4% in female prisoners in this study); however, the prevalence of attention-deficit hyper-activity disorder and other developmental or childhood disorders seems likely to have been under estimated.^57,58^ This underestimation might especially be the case for individuals released in the early 2000s who had shorter coverage of outpatient data than did individuals released later.

Another important limitation is that personality disorder was probably underestimated in this study. The proportion of patients with the disorder in this study contrasts with findings from investigations that use structured instruments, which, despite very high heterogeneity between primary studies, report prevalences of more than 50% in male and about 40% in female prisoners.^59^ However, our estimates are similar to those of three large carefully done studies in both remand and sentenced populations in England and Wales of between 7% and 11%.^60–62^ This finding underscores a wider issue in personality disorder research of investigators using structured instruments reporting much higher prevalences than do those of clinically based investigations; these clinically based studies might more closely identify individuals with treatment needs than would structured instruments. The first implication of this underestimation is that we might have overstated the effect of other psychiatric disorders and substance use disorder on violent reoffending because these disorders are moderated by personality disorder. We think that this overestimation is unlikely because research in the general population has shown that comorbid personality disorder does not explain the associations between other psychiatric disorders and violent crime.^63^ Additionally, our sibling models partly adjust for personality disorder. A second implication is that our PAFs are overestimates because they do not fully include all possible risk factors for violent reoffending. At the same time, PAFs provide an indication of the possible effect of treatment of a risk factor on population estimates of violent reoffending, and the evidence base of effective treatment for personality disorders is weak, at least in the prison setting.^64,65^

A further limitation is that the study was done in one country. Although the prison population is small in Sweden,^1^ some key characteristics of prisoners in Sweden are not very different from those in other high-income countries (eg, prevalence of psychiatric disorders, proportion of prisoners reoffending, length of incarceration; appendix p 12). Nevertheless, the extent to which our findings can be generalised to other countries needs further research. Because Sweden has a well-developed public health system (similar to that of the UK, but more accessible than that of the USA), our findings are likely to be on the conservative side in terms of estimation of the effects of psychiatric disorders in the international context, and the association between psychiatric disorders and violent reoffending might be even stronger in countries with less resourced prison health services. Finally, because we have used registers, we have information about a restricted set of covariates. A complex set of risk factors is likely to be implicated in reoffending, with different factors acting at different points, some of which will be proximal and unaccounted for in registers.

Many individuals with psychiatric disorders revolve between admission to hospital, homelessness, and the criminal justice system. Our findings underscore the need for improved detection, treatment, and management of prisoners with mental health disorders, and linkage of these prisoners to community-based mental care services on release.^66^ They also emphasise the need for further research into the role of psychiatric diagnoses in risk assessment for future offences and the effectiveness of diversion from criminal justice. Because the worldwide number of prisoners with psychiatric disorders is large, improvements to their treatment and management in custody and on release have the potential to improve their quality of life and counteract the cycle of reoffending.^67^

## Supplementary Material

Supplementary Appendix

## Figures and Tables

**Table 1:** Baseline sociodemographic and criminological information, and follow-up data for released prisoners in Sweden

	Men	Women
Number of individuals	43 840	3486
Number of person-years at risk	139 260	11 243
Incidents of violent reoffending during follow-up	10 884 (25%)	379 (11%)
Age group (years)		
16–24	8466 (19%)	361 (10%)
25–39	17 291 (39%)	1409 (40%)
≥40	18 083 (41%)	1716 (49%)
Civil status		
Unmarried	26 910 (65%)	1614 (49%)
Married	5066 (12%)	537 (16%)
Divorced	9105 (22%)	1094 (33%)
Widowed	222 (<1%)	67 (2%)
Highest length of education (years)		
<9	19 546 (47%)	1765 (53%)
9–11	19 174 (46%)	1322 (40%)
≥12	2583 (6%)	225 (7%)
Employed	8045 (20%)	355 (11%)
Immigrant	13 710 (31%)	806 (23%)
Disposable income (×100 Swedish Krona)	775 (473 to 1100)	749 (444 to 1082)
Neighbourhood deprivation*	0·38 (−0·17 to 1·48)	0·35 (−0·15 to 1·46)
Length of incarceration (months)		
<6	30 155 (69%)	2608 (75%)
6–11	7270 (17%)	506 (15%)
12–23	4408 (10%)	283 (8%)
≥24	2007 (5%)	89 (3%)
Violent index offence	17 294 (39%)	643 (18%)
Previous violent crime	23 960 (55%)	1112 (32%)
Previous psychiatric disorder		
Any psychiatric disorder	18 563 (42%)	2233 (64%)
Alcohol use disorder	9276 (21%)	968 (28%)
Drug use disorder	9597 (22%)	1438 (41%)
Personality disorder	2320 (5%)	353 (10%)
Attention-deficit hyperactivity disorder	546 (1%)	51 (1%)
Other developmental or childhood disorder	979 (2%)	139 (4%)
Schizophrenia spectrum disorders	1237 (3%)	130 (4%)
Bipolar disorder	216 (<1%)	35 (1%)
Depression	2553 (6%)	418 (12%)
Anxiety disorder	3247 (7%)	534 (15%)

Data are n, n (%), or median (IQR). 2573 men and 174 women had missing values for civil status, highest length of education, employment, disposable income, and neighbourhood deprivation.

* Standardised score of the overall degree of socioeconomic deprivation in an individual’s residential area.

**Table 2:** Association between any psychiatric disorder and violent crime reoffending

	Number of person-years at risk	Number of violent reoffences	Model 1*	Model 2†	Model 3‡
**Men**					
With psychiatric disorder	50 904	5658	2·10 (2·02–2·19)	1·63 (1·57–1·70)	2·01 (1·66–2·43)§
Without psychiatric disorder	88 356	5226	1	1	1
**Women**					
With psychiatric disorder	6595	301	2·76 (2·15–3·55)	2·02 (1·54–2·63)	0·52 (0·08–3·15)¶
Without psychiatric disorder	4648	78	1	1	1

Data are n or hazard ratio (95% CI).

* Adjusted for age and immigration status.

† Adjusted for age, immigration status, and sociodemographic and criminological covariates.

‡ Fixed-effect sibling model, adjusted for all factors shared by siblings and measured covariates adjusted for in models 1 and 2.

§ Based on 1417 pairs of male prisoners who were full siblings.

¶ Based on 41 pairs of female prisoners who were full siblings.

**Table 3:** Association between individual psychiatric disorders and violent crime reoffending

	Model 1*	Model 2†	Model 3‡
**Men**			
Alcohol use disorder	2·14 (2·05–2·24)	1·63 (1·56–1·71)	1·45 (1·38–1·53)
Drug use disorder	2·13 (2·05–2·22)	1·65 (1·58–1·72)	1·52 (1·45–1·59)
Personality disorder	2·29 (2·14–2·45)	1·64 (1·53–1·76)	1·30 (1·21–1·40)
Attention-deficit hyperactivity disorder	2·22 (1·89–2·61)	1·56 (1·31–1·85)	1·31 (1·10–1·55)
Other developmental or childhood disorder	1·82 (1·65–2·01)	1·46 (1·32–1·61)	1·33 (1·20–1·47)
Schizophrenia spectrum disorders	2·06 (1·87–2·26)	1·51 (1·37–1·67)	1·20 (1·09–1·33)
Bipolar disorder	1·96 (1·50–2·58)	1·75 (1·32–2·32)	1·50 (1·13–1·99)
Depression	1·41 (1·30–1·54)	1·28 (1·18–1·40)	1·09 (1·00–1·18)
Anxiety disorder	1·41 (1·32–1·51)	1·23 (1·14–1·32)	1·09 (1·01–1·17)
**Women**			
Alcohol use disorder	2·65 (2·15–3·26)	2·08 (1·66–2·60)	1·84 (1·46–2·32)
Drug use disorder	2·59 (2·10–3·20)	1·84 (1·46–2·30)	1·58 (1·26–2·00)
Personality disorder	2·57 (1·99–3·33)	1·66 (1·27–2·18)	1·27 (0·96–1·68)
Attention-deficit hyperactivity disorder	2·01 (0·95–4·25)	1·53 (0·72–3·27)	1·20 (0·56–2·57)
Other developmental or childhood disorder	1·84 (1·29–2·64)	1·20 (0·82–1·76)	1·04 (0·70–1·53)
Schizophrenia spectrum disorders	1·75 (1·11–2·74)	1·04 (0·64–1·69)	0·74 (0·45–1·20)
Bipolar disorder	2·84 (1·06–7·65)	1·81 (0·67–4·91)	1·35 (0·49–3·68)
Depression	1·49 (1·11–2·00)	1·36 (1·00–1·86)	1·16 (0·85–1·59)
Anxiety disorder	1·40 (1·07–1·83)	1·21 (0·92–1·60)	1·07 (0·81–1·41)

Data are hazard ratio (95% CI).

* Adjusted for age and immigration status.

† Adjusted for age, immigration status, and sociodemographic and criminological covariates.

‡ Adjusted for age, immigration status, sociodemographic and criminological covariates, and alcohol and drug use disorders.

**Table 4:** Violent reoffending in male prisoners with psychiatric disorder with and without substance use disorder comorbidity

	**Incidents of violent reoffending**		Adjusted hazard ratio*		
	Without substance use disorder	With substance use disorder	Without substance use disorder	With substance use disorder	p value for interaction†
Any psychiatric disorder‡	764/3426 (22%)	1912/5504 (35%)	1·39 (1·29–1·50)	2·43 (2·30–2·57)	0·85
Schizophrenia spectrum disorder	66/303 (22%)	390/934 (42%)	1·29 (1·00–1·67)	2·68 (2·41–2·98)	0·44
Bipolar disorder	11/72 (15%)	41/144 (28%)	1·45 (0·75–2·79)	3·22 (2·35–4·39)	0·67

Data are n/N (%) or hazard ratio (95% CI).

* Compared with prisoners without any psychiatric disorder, adjusted for age, immigration status, and sociodemographic and criminological covariates.

† Between any psychiatric disorder and substance use disorder.

‡ Excluding substance use disorder.

## Research in context

### Evidence before this study

We searched PubMed from Jan 1, 2004, to Dec 31, 2014, using the search terms “psychiatric disorder*”, “mental disorder*”, “mental illness*”, “recidi*”, “reoffend*”, and “repeat offend*”, with no language restrictions. We identified one systematic review on the risk of repeat offending in individuals with psychotic disorders and a further 13 studies since publication of that review (appendix pp 16–18). Although authors of one review found some evidence in support of psychotic disorders increasing reoffending risk, only one included study looked at prisoners and none studied violent reoffending. In these 13 newer studies, findings were inconsistent, with those from eight studies showing no independent association between psychiatric disorders and reoffending. However, seven of these studies were small or in selected samples, and the remaining one did not account for important sociodemographic confounders. Furthermore, we identified no studies that considered the effect of familial factors on the link between psychiatric disorders and reoffending. We identified one systematic review on personality disorders and repeat offending and one review on risk of recidivism for offenders with mental disorders. Authors of the first noted that personality disorders were consistently associated with an increased risk of reoffending, with low heterogeneity between these primary reports and little difference between all personality disorders and antisocial personality disorder. Authors of the second, which included heterogeneous samples, concluded that antisocial personality disorder predicts recidivism.

### Added value of this study

We are the first, to our knowledge, to investigate the association between psychiatric disorders and violent reoffending while taking into account both measured (sociodemographic and criminological) and unmeasured (familial) confounding factors. Use of a total population cohort of released prisoners enabled us to provide precise effect sizes and estimate the possible population impact of psychiatric disorders on violent reoffending. We found that some psychiatric disorders were associated with a substantially increased hazard of violent reoffending. We reported some evidence for potentially important heterogeneity between individual diagnoses and risk of violent reoffending.

### Implications of all the available evidence

Our findings underscore the need for improved detection, treatment, and management of prisoners with psychiatric and substance use disorders, and linkage of these prisoners to community-based mental health services on release. Further research into the role of psychiatric diagnoses in violent risk assessment and the effectiveness of diversion from criminal justice is needed.
