# Supplementary material for: Psychiatric disorders and violent reoffending: a national cohort study of convicted prisoners in Sweden
Source: Lancet Psychiatry. Author manuscript; Available in PMC 2015 Nov 2. (PMC4629414; doi:10.1016/S2215-0366(15)00234-5)
Supplement: Supplementary Appendix [file NIHMS65384-supplement-Supplementary_Appendix.pdf]

THE LANCET Psychiatry

Supplementary appendix

This appendix formed part of the original submission and has been peer reviewed. We post it as supplied by the authors.

Supplement to: Chang Z, Larsson H, Lichtenstein P, Fazel S. Psychiatric disorders and violent reoffending: a national cohort study of convicted prisoners in Sweden. *Lancet Psychiatry* 2015; published online Sept 3. http://dx.doi.org/10.1016/S2215-0366(15)00234-5.

Supplementary Figure 1. Kaplan–Meier Curves for violent reoffending in released prisoners in Sweden.

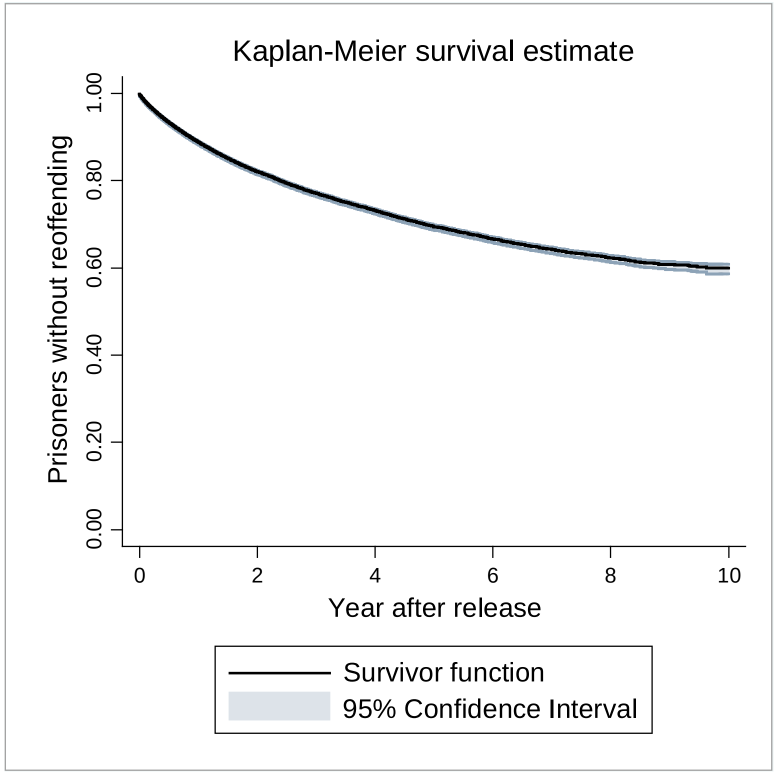

Supplementary Table 1. First violent reoffending after prison release.

Type of violent crime	Men (n, % of first violent reoffending)	Female (n, % of first violent reoffending)
Homicide	124 (1.1%)	3 (0.8%)
Assault	6893 (63.3%)	278 (73.4%)
Sexual offenses	270 (2.5%)	0 (0%)
Robbery	961 (8.8%)	19 (5.0%)
Arson	59 (0.6%)	5 (1.3%)
Threats & intimidation*	4000 (36.8%)	112 (29.6%)

* A person who raises a weapon against another or otherwise threatens to commit a criminal act, in such a manner that the nature thereof evokes in the threatened person a serious fear for the safety of his own or someone else's person or property, shall be sentenced for unlawful threat to a fine or imprisonment for at most one year. A person who physically molests or by discharging a firearm, throwing stones, making loud noise or other reckless conduct molests another, shall be sentenced for molestation to a fine or imprisonment for at most one year. If the crime is gross, imprisonment for at least six months and at most four years shall be imposed.

Supplementary Table 2. Association between Socio-demographic/clinical factors and violent reoffending in men and women.

	Number of individuals	Number of violent reoffending	Hazard ratio (95% CI)
Men			
Age group			
16-24 yr	8466	3259	1
25-39 yr	17 291	4637	0.54 (0.52-0.57)
>=40 yr	18 083	2988	0.34 (0.32-0.54)
Civil status			
Unmarried	26 910	7907	1
Married	5066	754	0.45 (0.42-0.49)
Divorced	9105	7657	0.56 (0.53-0.59)
Widowed	222	24	0.31 (0.21-0.46)
Highest education			
<= 9 yr	19 546	6033	1
9-12 yr	19 174	4049	0.62 (0.60-0.65)
> 12yr	2583	260	0.30 (0.26-0.34)
Employed			
Yes	8045	1140	0.44 (0.41-0.47)
No	24 047	9202	1
Immigrant			
Yes	13 710	3041	0.88 (0.85-0.92)
No	30 130	7843	1
Length of incarceration			
<= 6 mo	30 155	8091	1
6-12 mo	7270	1764	0.99 (0.95-1.05)
12-24 mo	4408	783	0.79 (0.74-0.85)
>=24 mo	2007	246	0.74 (0.65-0.85)
Violent index offence			
Yes	17 294	5501	1.80 (1.74-1.87)
No	26 546	5383	1
Previous violent crime			

Yes	23 960	8406	3.14 (3.00-3.29)
No	19 880	2478	1
Any psychiatric disorder			
Yes	18 563	5658	1.80 (1.73-1.87)
No	25 277	5226	1
Alcohol abuse			
Yes	9276	2888	1.48 (1.42-1.54)
No	34 564	7996	1
Drug abuse			
Yes	9597	3547	2.10 (2.01-2.18)
No	34 243	7337	1
Personality disorder			
Yes	2320	950	1.94 (1.82-2.08)
No	41 520	9934	1
ADHD			
Yes	546	151	3.21 (2.73-3.77)
No	43 294	10 733	1
Other developmental or childhood disorder			
Yes	979	425	2.41 (2.18-2.65)
No	42 861	10 459	1
Schizophrenia spectrum disorder			
Yes	1237	456	1.83 (1.66-2.00)
No	42 603	10 428	1
Bipolar disorder			
Yes	216	52	1.60 (1.22-2.1)
No	43 624	10 832	1
Depression			
Yes	2553	576	1.18 (1.09-1.28)
No	41 287	10 308	1
Anxiety disorder			
Yes	3247	892	1.33 (1.25-1.43)
No	40 593	9992	1

	Number of individuals	Number of violent reoffending	Hazard ratio (95% CI)
Women			
Age group			
16-24 yr	361	63	1
25-39 yr	1409	197	0.73 (0.55-0.96)
>=40 yr	1716	119	0.37 (0.27-0.50)
Civil status			
Unmarried	1614	216	1
Married	537	41	0.52 (0.37-0.72)
Divorced	1094	95	0.60 (0.46-0.74)
Widowed	67	5	0.54 (0.22-1.32)
Highest education			
<= 9 yr	1765	232	1
9-12 yr	1322	116	0.64 (0.52-0.81)
> 12yr	225	9	0.31 (0.16-0.60)
Employed			
Yes	355	9	0.18 (0.09-.35)
No	2957	348	1
Immigrant			
Yes	806	82	0.84 (0.66-1.08)
No	2680	297	1
Length of incarceration			
<= 6 mo	2608	309	1
6-12 mo	506	48	0.70 (0.51-0.94)
12-24 mo	283	18	0.47 (0.29-0.75)
>=24 mo	89	4	0.48 (0.18-1.23)
Violent index offence			
Yes	643	115	2.14 (1.72-2.67)
No	2843	264	1
Previous violent crime			
Yes	1112	228	3.66 (2.98-4.5)
No	2374	151	1

Any psychiatric disorder			
Yes	2233	301	2.72 (2.12-3.49)
No	1253	78	1
Alcohol abuse			
Yes	968	163	2.18 (1.78-2.67)
No	2518	216	1
Drug abuse			
Yes	1438	235	2.76 (2.24-3.40)
No	2048	144	1
Personality disorder			
Yes	353	73	2.79 (2.16-3.60)
No	3133	306	1
ADHD			
Yes	51	7	2.37 (1.12-5.01)
No	3435	372	1
Other developmental or childhood disorder			
Yes	139	34	2.49 (1.75-3.54)
No	3347	345	1
Schizophrenia spectrum disorder			
Yes	130	20	1.77 (1.13-2.78)
No	3356	359	1
Bipolar disorder			
Yes	35	4	2.10 (0.78-5.62)
No	3451	375	1
Depression			
Yes	418	51	1.41 (1.05-1.89)
No	3086	328	1
Anxiety disorder			
Yes	534	66	1.37 (1.05-1.79)
No	2952	313	1

Supplementary Table 3. Association between any psychiatric disorder and violent crime in released prisoners and their siblings (Hazard ratios with 95% confidence intervals).

	Prisoner	Psychiatric disorder	Number of individuals	Number of violent crimes	Adjusted model [a]	Fixed-effect sibling model [b]
Male	+	+	7531	2263	5·43 (4·79-6·16)	9·60 (7·41-12·4)
	+	-	9365	2012	3·27 (2·90-3·69)	4·77 (3·84-5·93)
	-	+	3083	217	2·26 (1·91-2·66)	2·31 (1·85-2·88)
	-	-	18 088	577	1	1
Female	+	+	854	106	4·85 (2·39-9·84)	3·39 (0·71-16·3)
	+	-	435	31	2·45 (1·13-5·31)	6·49 (1·03-40·8)
	-	+	349	9	1·87 (0·77-4·55)	0·92 (0·21-4·03)
	-	-	1442	18	1	1

[a] Adjusted for age and immigration status, socio-demographic and criminological covariates, with robust standard errors accounting for the correlation between siblings.

[b] Adjusted for all factors shared by siblings and measured covariates.

Supplementary Table 4. Association between any psychiatric disorder and violent reoffending in male prisoners, using different age cohorts.

	Psychiatric disorder	Number of individuals	Number of reoffending	Model 1 [a]	Model 2 [b]
Age at release					
<25 years	with	2674	1158	1·73 (1·61-1·86)	1·45 (1·35-1·57)
	without	5792	2101	1	1
>= 25 years	with	15 889	4500	2·32 (2·21-2·43)	1·72 (1·64-1·81)
	without	19 485	3125	1	1

[a] Adjusted for age and immigration status.

[b] Adjusted for age and immigration status, socio-demographic and criminological covariates.

Supplementary Table 5. Association between any psychiatric disorder and violent reoffending, using different outcome definitions.

	Psychiatric disorder	Number of individuals	Number of reoffending	Model 1 [a]	Model 2 [b]
Male					
Interpersonal violent crime [c]	with	18 563	4037	2.06 (1.97-2.16)	1.61 (1.53-1.68)
	without	25 277	3999	1	1
Any other violent crime [d]	with	18 563	2322	2.25 (2.11-2.40)	1.73 (1.62-1.85)
	without	25 277	1838	1	1
Female					
Interpersonal violent crime	with	2233	238	2.69 (2.03-3.58)	1.94 (1.44-2.63)
	without	1253	62	1	1
Any other violent crime	with	2233	91	3.02 (1.89-4.83)	2.11 (1.30-3.44)
	without	1253	26	1	1

[a] Adjusted for age and immigration status.

[b] Adjusted for age and immigration status, socio-demographic and criminological covariates.

[c] Including homicide and attempted homicide, all forms of assault (including aggravated, and assault of an officer), rape, sexual coercion, and child molestation.

[d] Including arson, indecent exposure, sexual harassment, illegal threats, and intimidation.

Supplementary Table 6. Association between individual psychiatric disorders and reoffending of interpersonal violent crime in male prisoners (Hazard ratios with 95% confidence intervals).

	Model 1 [a]	Model 2 [b]	Model 3 [c]
**Male**			
Alcohol abuse	2.15 (2.04-2.27)	1.65 (1.56-1.75)	1.47 (1.39-1.56)
Drug abuse	2.13 (2.03-2.24)	1.66 (1.58-1.74)	1.52 (1.44-1.60)
Personality disorder	2.19 (2.02-2.37)	1.58 (1.46-1.72)	1.24 (1.14-1.35)
ADHD	2.17 (1.80-2.62)	1.57 (1.29-1.92)	1.32 (1.08-1.60)
Other developmental or childhood disorder	1.77 (1.58-1.98)	1.43 (1.27-1.61)	1.31 (1.16-1.47)
Schizophrenia spectrum disorder	2.05 (1.83-2.29)	1.50 (1.34-1.68)	1.19 (1.06-1.33)
Bipolar disorder	1.80 (1.29-2.53)	1.63 (1.15-2.31)	1.39 (0.98-1.97)
Depression	1.39 (1.26-1.54)	1.27 (1.15-1.41)	1.07 (0.97-1.19)
Anxiety disorder	1.33 (1.23-1.45)	1.16 (1.06-1.26)	1.02 (0.94-1.11)

Supplementary Table 7. Association between individual psychiatric disorders and reoffending of other violent crime in male prisoners (Hazard ratios with 95% confidence intervals).

	Model 1 [a]	Model 2 [b]	Model 3 [c]
**Male**			
Alcohol abuse	2.20 (2.05-2.37)	1.66 (1.54-1.80)	1.48 (1.37-1.60)
Drug abuse	2.17 (2.03-2.32)	1.66 (1.55-1.78)	1.52 (1.41-1.63)
Personality disorder	2.41 (2.17-2.68)	1.69 (1.52-1.89)	1.33 (1.19-1.49)
ADHD	2.40 (1.84-3.14)	1.59 (1.19-2.13)	1.33 (0.99-1.77)
Other developmental or childhood disorder	2.06 (1.76-2.41)	1.62 (1.37-1.90)	1.47 (1.24-1.73)
Schizophrenia spectrum disorder	2.13 (1.83-2.47)	1.54 (1.32-1.80)	1.22 (1.04-1.43)
Bipolar disorder	2.15 (1.42-3.28)	1.89 (1.23-2.91)	1.62 (1.05-2.49)
Depression	1.57 (1.38-1.79)	1.41 (1.23-1.61)	1.19 (1.04-1.36)
Anxiety disorder	1.62 (1.45-1.80)	1.40 (1.25-1.56)	1.24 (1.11-1.38)

Supplementary Table 8. Summary of key characteristics of prisoners in Sweden, the US, and England and Wales.

	Sweden	US	UK (England & Wales)
Exposure			
Alcohol abuse (men)	21%	26% [a]	21% [a]
Alcohol abuse (women)	28%	20% [a]	n/a
Drug abuse (men)	22%	25% [a]	39% [a]
Drug abuse (women)	41%	45% [a]	n/a
Psychosis (men)	3%	3% [b]	4% [c]
Psychosis (women)	4%	3% [b]	4% [c]
Outcome			
2-year reconviction	43% [d]	36% [e]	n/a [f]
Prison Sentence			
Length of incarceration less than or equal to 6 months	69%	66% [g]	52% [h]
Prisoner population			
Minority ethnic groups	30%	39%/21% [i]	26% [j]

[a] Fazel S, Bains P, Doll H. Substance abuse and dependence in prisoners: a systematic review. Addiction 2006; 101(2): 181-91.
[b] Baillargeon J, Binswanger IA, Penn JV, Williams BA, Murray OJ. Psychiatric disorders and repeat incarcerations: the revolving prison door. The American journal of psychiatry 2009; 166(1): 103-9.
[c] Pooled prevalence. Fazel S, Seewald K. Severe mental illness in 33,588 prisoners worldwide: systematic review and meta-regression analysis. The British journal of psychiatry : the journal of mental science 2012; 200(5): 364-73.
[d] Graunbøl HM, Kielstrup B, Muiluvuori M-L, et al. Retur: en nordisk undersøgelse af recidiv blant klienter i kriminalforsorgen: Kriminalomsorgens utdanningssenter; 2010.
[e] Langan P, Levin D. Recidivism of Prisoners Released in 1994. Washington, DC: Bureau of Justice Statistics; 2002.
[f] Unlike other counties, the reported reoffending (59% in 2 years) in England and Wales includes fines.
[g] Binswanger IA, Blatchford PJ, Mueller SR, Stern MF. Mortality after prison release: opioid overdose and other causes of death, risk factors, and time trends from 1999 to 2009. Annals of internal medicine 2013; 159(9): 592-600.
[h] Ministry of Justice. Offender management annual tables 2013. 2014. London: Ministry of Justice.
[i] Black/Hispanic. Bureau of Justice Statistics. Prison Inmates at Midyear 2009 - Statistical Tables. 2010.
[j] Prison Reform Trust. Bromley Briefings Prison Factfile Summer 2014. 2014.

STROBE Statement—checklist of items that should be included in reports of observational studies

	Item No	Recommendation	Page #
**Title and abstract**	1	(*a*) Indicate the study's design with a commonly used term in the title or the abstract	1
		(*b*) Provide in the abstract an informative and balanced summary of what was done and what was found	2
**Introduction**			
Background/rationale	2	Explain the scientific background and rationale for the investigation being reported	3-4
Objectives	3	State specific objectives, including any prespecified hypotheses	4
**Methods**			
Study design	4	Present key elements of study design early in the paper	4
Setting	5	Describe the setting, locations, and relevant dates, including periods of recruitment, exposure, follow-up, and data collection	4
Participants	6	(*a*) *Cohort study*—Give the eligibility criteria, and the sources and methods of selection of participants. Describe methods of follow-up<br>*Case-control study*—Give the eligibility criteria, and the sources and methods of case ascertainment and control selection. Give the rationale for the choice of cases and controls<br>*Cross-sectional study*—Give the eligibility criteria, and the sources and methods of selection of participants	4-5
		(*b*) *Cohort study*—For matched studies, give matching criteria and number of exposed and unexposed<br>*Case-control study*—For matched studies, give matching criteria and the number of controls per case	--
Variables	7	Clearly define all outcomes, exposures, predictors, potential confounders, and effect modifiers. Give diagnostic criteria, if applicable	4-5
Data sources/ measurement	8*	For each variable of interest, give sources of data and details of methods of assessment (measurement). Describe comparability of assessment methods if there is more than one group	4-5
Bias	9	Describe any efforts to address potential sources of bias	6-7
Study size	10	Explain how the study size was arrived at	4
Quantitative variables	11	Explain how quantitative variables were handled in the analyses. If applicable, describe which groupings were chosen and why	5
Statistical methods	12	(*a*) Describe all statistical methods, including those used to control for confounding	6
		(*b*) Describe any methods used to examine subgroups and interactions	6-7
		(*c*) Explain how missing data were addressed	6-7
		(*d*) *Cohort study*—If applicable, explain how loss to follow-up was addressed<br>*Case-control study*—If applicable, explain how matching of cases and controls was addressed<br>*Cross-sectional study*—If applicable, describe analytical methods taking account of sampling strategy	4,6
		(*e*) Describe any sensitivity analyses	7

**Results**			
Participants	13*	(a) Report numbers of individuals at each stage of study—eg numbers potentially eligible, examined for eligibility, confirmed eligible, included in the study, completing follow-up, and analysed	7, 21
		(b) Give reasons for non-participation at each stage	--
		(c) Consider use of a flow diagram	--
Descriptive data	14*	(a) Give characteristics of study participants (eg demographic, clinical, social) and information on exposures and potential confounders	7, 21
		(b) Indicate number of participants with missing data for each variable of interest	21
		(c) *Cohort study*—Summarise follow-up time (eg, average and total amount)	7, 21
Outcome data	15*	*Cohort study*—Report numbers of outcome events or summary measures over time	7, 21
		Case-control study—Report numbers in each exposure category, or summary measures of exposure	
		Cross-sectional study—Report numbers of outcome events or summary measures	
Main results	16	(*a*) Give unadjusted estimates and, if applicable, confounder-adjusted estimates and their precision (eg, 95% confidence interval). Make clear which confounders were adjusted for and why they were included	8
		(*b*) Report category boundaries when continuous variables were categorized	--
		(*c*) If relevant, consider translating estimates of relative risk into absolute risk for a meaningful time period	--
Other analyses	17	Report other analyses done—eg analyses of subgroups and interactions, and sensitivity analyses	9
**Discussion**			
Key results	18	Summarise key results with reference to study objectives	9
Limitations	19	Discuss limitations of the study, taking into account sources of potential bias or imprecision. Discuss both direction and magnitude of any potential bias	11-12
Interpretation	20	Give a cautious overall interpretation of results considering objectives, limitations, multiplicity of analyses, results from similar studies, and other relevant evidence	9-11
Generalisability	21	Discuss the generalisability (external validity) of the study results	12
**Other information**			
Funding	22	Give the source of funding and the role of the funders for the present study and, if applicable, for the original study on which the present article is based	7

*Give information separately for cases and controls in case-control studies and, if applicable, for exposed and unexposed groups in cohort and cross-sectional studies.

Note: An Explanation and Elaboration article discusses each checklist item and gives methodological background and published examples of transparent reporting. The STROBE checklist is best used in conjunction with this article (freely available on the Web sites of PLoS Medicine at http://www.plosmedicine.org/, Annals of Internal Medicine at http://www.annals.org/, and Epidemiology at http://www.epidem.com/). Information on the STROBE Initiative is available at www.strobe-statement.org

Supplementary information for the Panel: Research in context

Figure: Systematic search process and included studies.

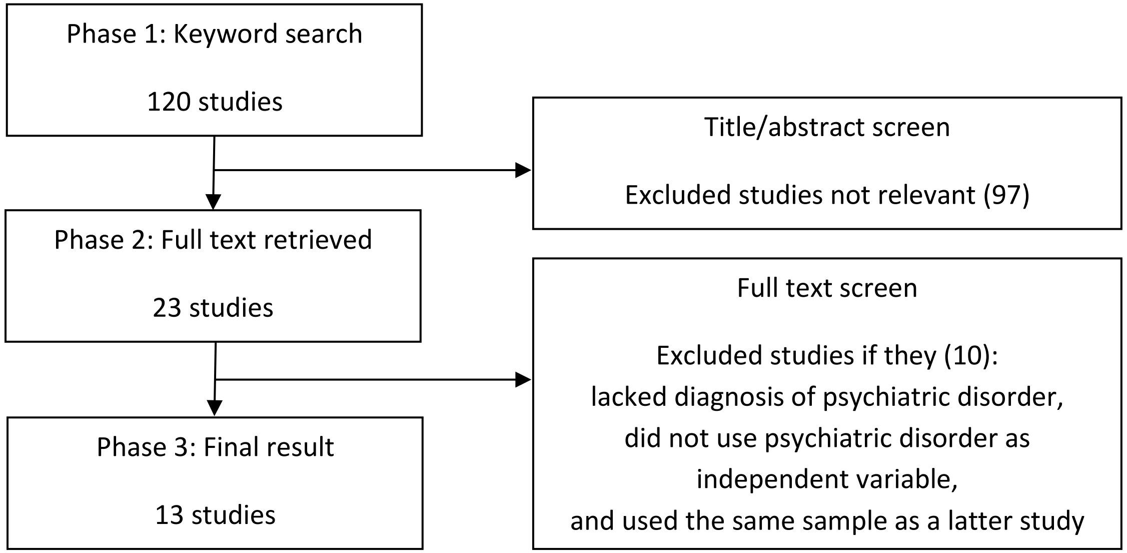

Table. Characteristics of the studies included in the review of psychiatric disorders and reoffending.

Study Name	Region	Sample Size	Gender	Setting	Results
Abracen et al, 2014 [1]	Canada	136	Male	Paroled offenders	Men with borderline personality disorder were more likely to reoffend (general and violent) than those without. Other psychiatric disorders were not associated with reoffending in the adjusted model.
Baillargeon et al, 2010 [2]	United States	61,248	Mixed	Prisoners	Prisoners with a co-occurring psychiatric and substance use disorder exhibited a higher risk of multiple incarcerations compared to inmates with psychiatric disorders alone or substance use disorders alone.
Cloyes et al, 2010 [3]	United States	9,245	Mixed	Prisoners	Women with serious mental illness were at increased risk of repeated incarceration.
Colins et al, 2011 [4]	Belgium	232	Male	Detained adolescents	Common psychiatric disorders did not significantly increase the risk of subsequent arrests, with the exception that substance use disorders increased the risk of later substance-related recidivism.
Du et al, 2013 [5]	United States	1,444	Mixed	Drug-abusing offenders	No specific type of mental disorder seemed to be predictive of recidivism.
Fu et al, 2013 [6]	United States	798	Mixed	HIV-infected jail detainees	Diagnoses of a major psychiatric disorder were predictive of recidivism and associated with shorter time to re-incarceration.
Hawthorne et al, 2012 [7]	United States	4,356	Mixed	Jail detainees with psychiatric diagnoses	Diagnoses of schizophrenia or bipolar disorder were associated with higher risk of re-incarceration than major depression.
Lund et al, 2013 [8]	Sweden	163	Male	Offenders with mental disorder	People with substance abuse had higher rates of violent reconvictions than those with other disorders.
Skeem et al, 2014[9]	United States	221	Mixed	Parolees	Offenders with mental illness were equally likely to be rearrested, but were more likely to return to prison custody, compared with those without mental illness.
Sturup et al, 2014 [10]	Sweden	153	Mixed	Homicide offenders	Diagnoses of psychotic disorder were predictive of violent recidivism. Effects of personality disorder and addiction were not significant.
Walters & Crawford, 2014 [11]	United States	1,163	Male	Prisoners	Major mental illness was not predictive of recidivism.
Willging et al, 2013 [12]	United States	98	Female	Prisoners	Having co-occurring mental illness and substance dependence predicted re-incarceration, and mental illness or substance dependence alone did not.

Wilson et al, 2014 [13]	United States	16,434	Mixed	Jail inmates	People with serious mental illness alone were not at increased risk of re-incarceration. People with co-occurring disorders had a higher risk of re-incarceration than that of individuals with no diagnosis.

Reference

1. Abracen J, Langton CM, Looman J, et al. Mental Health Diagnoses and Recidivism in Paroled Offenders. *International journal of offender therapy and comparative criminology.* May 2 2013;58(7):765-779.
2. Baillargeon J, Penn JV, Knight K, Harzke AJ, Baillargeon G, Becker EA. Risk of reincarceration among prisoners with co-occurring severe mental illness and substance use disorders. *Administration and policy in mental health.* Jul 2010;37(4):367-374.
3. Cloyes KG, Wong B, Latimer S, Abarca J. Women, serious mental illness and recidivism: A gender-based analysis of recidivism risk for women with SMI released from prison. *Journal of forensic nursing.* Spring 2010;6(1):3-14.
4. Colins O, Vermeiren R, Vahl P, Markus M, Broekaert E, Doreleijers T. Psychiatric disorder in detained male adolescents as risk factor for serious recidivism. *Canadian journal of psychiatry. Revue canadienne de psychiatrie.* Jan 2011;56(1):44-50.
5. Du J, Huang D, Zhao M, Hser YI. Drug-abusing offenders with co-morbid mental disorders: gender differences in problem severity, treatment participation, and recidivism. *Biomedical and environmental sciences : BES.* Jan 2013;26(1):32-39.
6. Fu JJ, Herme M, Wickersham JA, et al. Understanding the revolving door: individual and structural-level predictors of recidivism among individuals with HIV leaving jail. *AIDS and behavior.* Oct 2013;17 Suppl 2:S145-155.
7. Hawthorne WB, Folsom DP, Sommerfeld DH, et al. Incarceration among adults who are in the public mental health system: rates, risk factors, and short-term outcomes. *Psychiatric services (Washington, D.C.).* Jan 2012;63(1):26-32.
8. Lund C, Hofvander B, Forsman A, Anckarsater H, Nilsson T. Violent criminal recidivism in mentally disordered offenders: a follow-up study of 13-20 years through different sanctions. *International journal of law and psychiatry.* May-Aug 2013;36(3-4):250-257.
9. Skeem JL, Winter E, Kennealy PJ, Louden JE, Tatar JR, 2nd. Offenders with mental illness have criminogenic needs, too: toward recidivism reduction. *Law and human behavior.* Jun 2014;38(3):212-224.
10. Sturup J, Lindqvist P. Homicide offenders 32 years later - a Swedish population-based study on recidivism. *Criminal behaviour and mental health : CBMH.* Feb 2014;24(1):5-17.
11. Walters GD, Crawford G. Major mental illness and violence history as predictors of institutional misconduct and recidivism: main and interaction effects. *Law and human behavior.* Jun 2014;38(3):238-247.
12. Willging CE, Malcoe LH, St Cyr S, Zywiak WH, Lapham SC. Behavioral health and social correlates of reincarceration among Hispanic, Native American, and white rural women. *Psychiatric services (Washington, D.C.).* Jun 2013;64(6):590-593.
13. Blank Wilson A, Draine J, Barrenger S, Hadley T, Evans A, Jr. Examining the impact of mental illness and substance use on time till re-incarceration in a county jail. *Administration and policy in mental health.* May 2014;41(3):293-301.
